# The *ADAMTS9* gene is associated with cognitive aging in the elderly in a Taiwanese population

**DOI:** 10.1371/journal.pone.0172440

**Published:** 2017-02-22

**Authors:** Eugene Lin, Shih-Jen Tsai, Po-Hsiu Kuo, Yu-Li Liu, Albert C. Yang, Chung-Feng Kao, Cheng-Hung Yang

**Affiliations:** 1 Institute of Biomedical Sciences, China Medical University, Taichung, Taiwan; 2 Vita Genomics, Inc., Taipei, Taiwan; 3 TickleFish Systems Corporation, Seattle, WA, United States of America; 4 Department of Psychiatry, Taipei Veterans General Hospital, Taipei, Taiwan; 5 Division of Psychiatry, National Yang-Ming University, Taipei, Taiwan; 6 Department of Public Health, Institute of Epidemiology and Preventive Medicine, National Taiwan University, Taipei, Taiwan; 7 Center for Neuropsychiatric Research, National Health Research Institutes, Miaoli County, Taiwan; 8 Department of Agronomy, College of Agriculture & Natural Resources, National Chung Hsing University, Taichung, Taiwan; Kunming Institute of Zoology, Chinese Academy of Sciences, CHINA

## Abstract

Evidence indicates that the pathophysiologic mechanisms associated with insulin resistance may contribute to cognitive aging and Alzheimer’s diseases. In this study, we hypothesize that single nucleotide polymorphisms (SNPs) within insulin resistance-associated genes, such as the ADAM metallopeptidase with thrombospondin type 1 motif 9 (*ADAMTS9*), glucokinase regulator (*GCKR*), and peroxisome proliferator activated receptor gamma (*PPARG*) genes, may be linked with cognitive aging independently and/or through complex interactions in an older Taiwanese population. A total of 547 Taiwanese subjects aged over 60 years from the Taiwan Biobank were analyzed. Mini-Mental State Examinations (MMSE) were administered to all subjects, and MMSE scores were used to measure cognitive functions. Our data showed that four SNPs (rs73832338, rs9985304, rs4317088, and rs9831846) in the *ADAMTS9* gene were significantly associated with cognitive aging among the subjects (P = 1.5 x 10^−6^ ~ 0.0002). This association remained significant after performing Bonferroni correction. Additionally, we found that interactions between the *ADAMTS9* rs9985304 and *ADAMTS9* rs76346246 SNPs influenced cognitive aging (P < 0.001). However, variants in the *GCKR* and *PPARG* genes had no association with cognitive aging in our study. Our study indicates that the *ADAMTS9* gene may contribute to susceptibility to cognitive aging independently as well as through SNP-SNP interactions.

## Introduction

There is growing evidence that patients with type 2 diabetes (T2D) have an increased risk of developing Alzheimer's disease (AD), as reported in several epidemiologic studies [1, 2]. Furthermore, bioinformatics analysis based on genome wide association study (GWAS) data indicated that it is probable for T2D and AD to share similar genetic etiology and mechanisms [3, 4]. Insulin resistance is known to be a precursor of T2D because insulin resistance can lead to T2D over time. Therefore, some physiological and pathogenic mechanisms that are associated with insulin resistance may also play a key role in AD [5–8]. Among the genes involved in the development of insulin resistance are the ADAM metallopeptidase with thrombospondin type 1 motif 9 (*ADAMTS9*), glucokinase regulator (*GCKR*), and peroxisome proliferator activated receptor gamma (*PPARG*) genes.

The *ADAMTS9* gene is located on chromosome 3p14.1 and encodes a member of a disintegrin and metalloproteinase with thrombospondin motifs (ADAMTS) protein family that has been implicated in the cleavage of proteoglycans, the control of organ shape during development, and the inhibition of angiogenesis. Boesgaard et al. reported an association of the T2D risk allele (C) of *ADAMTS9* rs4607103 with a decrease in the insulin sensitivity of peripheral tissues [9]. A subsequent study by Trombetta *et al*. indicated that the *ADAMTS9* rs4607103 SNP may contribute to susceptibility to insulin resistance in Italian subjects [10]. The ADAMTS-9 protein encoded by the *ADAMTS9* gene, a member of the ADAMTS protein family, may also play a key role in the physiological and pathological mechanisms of central nervous system disorders involving neuroinflammation in the lesion core and neuroplasticity in surrounding tissues, such as ischemic stroke and spinal cord injury [11–13].

The *GCKR* gene, located on chromosome 2p23.3, encodes a regulatory protein that belongs to the sugar isomerase protein family and inhibits glucokinase in liver and pancreatic islet cells [14]. It has been observed that the glucokinase regulatory protein encoded by the *GCKR* gene is present in adult rat and human brains, including in the ventromedial and arcuate nuclei of the hypothalamus, suggesting its participation in glucose sensing in the central nervous system [15, 16]. Additionally, the *GCKR rs780094* SNP has been linked to predisposition to insulin resistance in Danish [14] and Japanese [17] populations. It has also been suggested that the *GCKR* nt 11216 polymorphism is involved in increased insulin secretion [18].

The *PPARG* gene is located on chromosome 3p25.2 and encodes the peroxisome proliferator activated receptor (PPAR) gamma protein, which functions in adipocyte differentiation and glucose homeostasis [19, 20]. In several studies, the *PPARG* rs1801282 SNP was identified to be associated with insulin sensitivity [21–23] and cognitive impairment [24]. It has also been demonstrated that variants (including rs2972164, rs11128598, rs17793951, rs1151996, rs1175541, and rs3856806) in the *PPARG* gene were likely to influence insulin sensitivity in a Mexican population [25]. Moreover, it has been observed that PPAR gamma agonists generated short-term favorable effects in patients with early AD and mild cognitive impairment, because they might regulate in-vitro processing of the amyloid precursor protein, which plays a central role in the pathophysiology of AD [26].

Based on the aforementioned considerations, it was speculated that insulin resistance and its relevant genes may play a key role in the development of cognitive aging. Thus, we hypothesized that insulin resistance-associated genes, namely the *ADAMTS9*, *GCKR*, and *PPARG* genes, may be linked with cognitive aging. To our knowledge, the influence of these genes on cognitive aging is sparse in terms of human data. Therefore, we conducted an association study to weigh the relationships between susceptibility to cognitive aging and SNPs within the *ADAMTS9*, *GCKR*, and *PPARG* genes. We also assessed the potential SNP-SNP effects of these associations on cognitive aging.

## Materials and methods

### Study population

This study incorporated Taiwanese subjects from the Taiwan Biobank, which gathered information and specimens from participants in recruitment centers across Taiwan [27–30]. The study cohort consisted of 547 participants. Inclusion criteria were individuals who were aged 60 years or over and self-reported as being of Taiwanese Han Chinese ancestry [29]. Participants with a history of cancer were excluded [29]. In addition, we excluded participants with T2D because the investigated genes have been related to T2D [9, 17, 25]. Ethical approval for the study was granted by the Institutional Review Board of the Taiwan Biobank before conducting the study (approval number: 201506095RINC). Each subject signed the approved informed consent form. All experiments were performed in accordance with relevant guidelines and regulations. Education was defined based on whether or not high school was attended.

### Cognitive assessment

Global cognitive assessment was performed using the 30-point Mini-Mental State Examination (MMSE), which includes questions based on the five domains of orientation, registration, attention and calculation, recall, and language. We analyzed MMSE as a continuous outcome, as well as according to categories based on previously defined MMSE thresholds [31]: MMSE score ≥ 24 (normal) and MMSE score < 24 (cognitive impairment).

### Genotyping

DNA was isolated from blood samples using a QIAamp DNA blood kit following the manufacturer’s instructions (Qiagen, Valencia, CA, USA). The quality of the isolated genomic DNA was evaluated using agarose gel electrophoresis, and the quantity was determined by spectrophotometry [32, 33]. SNP genotyping was carried out using the custom Taiwan BioBank chips and run on the Axiom Genome-Wide Array Plate System (Affymetrix, Santa Clara, CA, USA). The SNP panel covered 141 SNPs from the following three genes, namely the *ADAMTS9*, *GCKR*, and *PPARG* genes (S1 Table). Eleven SNPs were excluded from further analyses due to failure to achieve Hardy-Weinberg equilibrium (P < 0.05) or due to a genotyping call rate < 0.95. The genotyping results, including minor allele frequencies, P values for Hardy-Weinberg equilibrium, and genotyping call rates are shown in S1 Table. Additionally, tag SNPs were identified by PLINK [34] with a linkage disequilibrium (LD) value of r^2^ = 0.8 as a threshold.

### Statistical analysis

In this study, we weighed the association of the investigated SNP with MMSE scores by a general linear model using age, gender, and education as covariates [35]. The genotype frequencies were assessed for Hardy-Weinberg equilibrium using a χ^2^ goodness-of-fit test with 1 degree of freedom (i.e. the number of genotypes minus the number of alleles). Multiple testing was adjusted for by using the Bonferroni correction. The criterion for significance was set at P < 0.05 for all tests. Data are presented as the mean ± standard deviation.

To investigate SNP-SNP interactions, we leveraged the generalized multifactor dimensionality reduction (GMDR) method [36]. We tested two-way up to three-way interactions using 10-fold cross-validation. The GMDR software provided some output parameters, including the testing accuracy and empirical P values, to assess each selected interaction. Moreover, we provided age, gender, and education as covariates for SNP-SNP interaction models in our interaction analyses. Permutation testing obtained empirical P values of prediction accuracy as a benchmark based on 1,000 shuffles.

## Results

Table 1 describes the demographic and clinical characteristics of the study population, which included 547 subjects. The median MMSE score was 27 and the interquartile range was 25–29.

**Table 1:** Demographic and clinical characteristics of Taiwanese study subjects.

Characteristic	Overall
No. of subjects, n	547
Mean age ± SD, years	64.1±3.0
Female, %	50.63
Less than high school graduate, n	214
MMSE score, median (IQR)	27 (25–29)

IQR = interquartile range, MMSE = Mini-Mental State Examination, SD = standard deviation.

Data are presented as mean ± standard deviation.

All participants self-reported as being of Taiwanese Han Chinese ancestry.

First, we investigated the association between cognitive aging and three insulin resistance-related genes, namely the *ADAMTS9*, *GCKR*, and *PPARG* genes. Based on LD, we filtered SNPs and selected 96 tag SNPs (S2 Table). Among the 96 tag SNPs assessed in this study (S3 Table), there were 16 tag SNPs in the *ADAMTS9* and *PPARG* genes showing evidence of association (P < 0.05) with MMSE scores (Table 2).

**Table 2:** Linear regression models of associations between the MMSE scores and 16 tag SNPs within the *ADAMTS9* and *PPARG* genes, which have an evidence of association (P < 0.05).

						Additive model			Recessive model			Dominant model		
Gene	CHR	SNP	A1	A2	MAF	BETA	SE	P	BETA	SE	P	BETA	SE	P
*ADAMTS9*	3	rs17070967	C	T	0.077	-0.04	0.70	0.9574	0.05	1.41	0.9716	-0.72	0.32	0.0264
		rs1561988	A	G	0.496	-0.42	0.17	0.0138	-0.78	0.28	0.0054	-0.33	0.28	0.2316
		rs1014640	T	C	0.441	-0.51	0.17	0.0034	-1.00	0.30	0.0010	-0.31	0.26	0.2344
		rs17071042	C	A	0.076	0.59	0.99	0.5527	1.29	1.99	0.5170	-0.66	0.33	0.0448
		rs3935273	T	C	0.218	-0.31	0.31	0.3097	-0.42	0.61	0.4918	-0.58	0.25	0.0185
		rs76346246	C	T	0.070	0.03	1.40	0.9818	0.18	2.81	0.9499	-0.96	0.34	0.0047
		rs60073432	A	G	0.251	0.29	0.24	0.2344	0.33	0.48	0.4860	0.57	0.24	0.0184
		rs73832338	T	C	0.207	1.01	0.31	0.0012	1.78	0.62	0.0042	0.93	0.25	**0.0002**
		rs9985304	A	G	0.427	-0.74	0.17	**1.4 x 10**^**−5**^	-1.22	0.30	**4.5 x 10**^**−5**^	-0.74	0.26	0.0040
		rs4317088	C	T	0.490	-0.82	0.17	**1.5 x 10**^**−6**^	-1.15	0.28	**3.7 x 10**^**−5**^	-1.03	0.28	**0.0002**
		rs6445420	C	T	0.083	-1.45	0.63	0.0221	-2.87	1.26	0.0232	-0.39	0.35	0.2595
		rs9831846	C	T	0.484	-0.71	0.17	**2.2 x 10**^**−5**^	-1.03	0.27	**0.0002**	-0.88	0.27	0.0013
		rs7646362	A	G	0.046	0.83	1.40	0.5556	1.72	2.82	0.5408	-0.93	0.44	0.0353
		rs75581931	A	G	0.224	-0.68	0.31	0.0289	-1.32	0.61	0.0314	-0.22	0.24	0.3670
		rs80118777	G	T	0.150	-1.03	0.50	0.0397	-2.03	1.00	0.0424	-0.22	0.26	0.4000
*PPARG*	3	rs78287138	T	C	0.142	0.38	0.43	0.3703	0.62	0.86	0.4693	0.57	0.26	0.0314

A1 = minor allele, A2 = major allele, BETA = Beta coefficients, Chr = chromosome, HWE = Hardy–Weinberg equilibrium, MAF = minor allele frequency, MMSE = Mini-Mental State Examination, SE = standard error.

Analysis was obtained after adjustment for covariates including age, gender, and education. P values of < 0.0005 are shown in bold.

Furthermore, as shown in Table 2, the association with MMSE scores persisted with significance after applying Bonferroni correction (P < 0.05/96 = 0.0005) for four key SNPs in the *ADAMTS9* gene: rs73832338 (dominant model: P = 0.0002), rs9985304 (additive model: P = 1.4 x 10^−5^; recessive model: P = 4.5 x 10^−5^), rs4317088 (additive model: P = 1.5 x 10^−6^; recessive model: P = 3.7 x 10^−5^; dominant model: P = 0.0002), and rs9831846 (additive model: P = 2.2 x 10^−5^; recessive model: P = 0.0002). In addition, the rs9985304, rs4688490, rs6802863, and rs7636925 SNPs of the *ADAMTS9* gene were found to be in strong LD (r^2^ > 0.8) to each other (S2 Table). The *ADAMTS9* rs4317088 SNP was also in strong LD with the rs6784609, rs6802863, and rs9835360 SNPs (S2 Table).

Next, we employed categorized MMSE scores as an outcome (normal: MMSE score ≥ 24; cognitive impairment: MMSE score < 24) for SNP-SNP interaction analysis. The GMDR analysis was used to assess the impacts of combinations between the 96 tag SNPs in cognitive aging, including age, gender, and education as covariates. Table 3 summarizes the results obtained from GMDR analysis for two-way up to three-way SNP-SNP interaction models with covariate adjustment. As shown in Table 3, there was a significant two-way model involving *ADAMTS9* rs9985304 and *ADAMTS9* rs76346246 (P < 0.001), indicating a potential SNP-SNP interaction between *ADAMTS9* rs9985304 and *ADAMTS9* rs76346246 in influencing cognitive aging. Similarly, there was a significant three-way SNP-SNP interaction model involving *ADAMTS9* rs4317088, *ADAMTS9* rs4566532, and *ADAMTS9* rs7642530 (P < 0.001) in influencing cognitive aging.

**Table 3:** SNP-SNP interaction models identified by the GMDR method with adjustment for age, gender, and education.

Best interaction model	Testing accuracy (%)	P value
*ADAMTS9* rs9985304, *ADAMTS9* rs76346246	68.72	**< 0.001**
*ADAMTS9* rs4317088, *ADAMTS9* rs4566532, *ADAMTS9* rs7642530	67.40	**< 0.001**

GMDR = generalized multifactor dimensionality reduction.

P value was based on 1,000 permutations. Analysis was obtained after adjustment for covariates including age, gender, and education. P values of < 0.05 are shown in bold.

We further utilized multivariable logistic regression analysis with adjustment for age, gender, and education to assess the two-way *ADAMTS9* rs9985304 and *ADAMTS9* rs76346246 interaction models selected by the GMDR method (Table 4). Our analysis revealed that carriers with the AA genotype of *ADAMTS9* rs9985304 and the C allele of *ADAMTS9* rs76346246 had a 0.11-fold increased risk for cognitive aging, compared to those with the G allele of *ADAMTS9* rs9985304 and the TT genotype of *ADAMTS9* rs76346246 (Table 4). Carriers with the G allele of *ADAMTS9* rs9985304 and the C allele of *ADAMTS9* rs76346246 also had a 0.37-fold increased risk for cognitive aging, compared to those with the G allele of *ADAMTS9* rs9985304 and the TT genotype of *ADAMTS9* rs76346246 (Table 4). Finally, carriers with the AA genotype of *ADAMTS9* rs9985304 and the TT genotype of *ADAMTS9* rs76346246 had a 0.2-fold increased risk for cognitive aging, compared to those with the G allele of *ADAMTS9* rs9985304 and the TT genotype of *ADAMTS9* rs76346246 (Table 4).

**Table 4:** Multivariable logistic regression analysis for the *ADAMTS9* rs9985304 and *ADAMTS9* rs76346246 interaction model.

Two-way interaction model	OR	95% CI	P value^b^
*ADAMTS9* rs9985304 (GG+AG genotype) with *ADAMTS9* rs76346246 (TT genotype)^a^	1		
*ADAMTS9* rs9985304 (AA genotype) with *ADAMTS9* rs76346246 (CC+CT genotype)	0.11	0.03–0.36	**0.0003**
*ADAMTS9* rs9985304 (GG+AG genotype) with *ADAMTS9* rs76346246 (CC+CT genotype)	0.37	0.17–0.79	**0.0105**
*ADAMTS9* rs9985304 (AA genotype) with *ADAMTS9* rs76346246 (TT genotype)	0.20	0.11–0.37	**3.4 x 10**^**−7**^

CI = confidence interval, OR = odds ratio.

^a^ Reference.

^b^ Versus reference.

Analysis was obtained after adjustment for covariates including age, gender, and education. P values of < 0.05 are shown in bold.

To further study the association of SNP-age interactions with cognitive measures, we employed categorized MMSE scores as an outcome and utilized multivariable logistic regression analysis with adjustment for gender and education (Table 5). To stratify the data by age, two groups were defined using the median of age (Group I: individuals with age ≦ 64 years; Group II: individuals with age > 64 years). Our analysis revealed that the Group I subjects with the G allele of *ADAMTS9* rs9985304 had a 6.51-fold increased risk for cognitive aging, compared to those with the AA genotype of *ADAMTS9* rs9985304 (Table 5). Additionally, the Group II subjects with the GG+AG genotypes of *ADAMTS9* rs9985304 had a 2.67-fold increased risk for cognitive aging, compared to the Group I subjects with the AA genotype of *ADAMTS9* rs9985304 (Table 5). Our analysis also implicated the interaction effect between *ADAMTS9* rs73832338 and age (P = 0.0016), between *ADAMTS9* rs4317088 and age (P = 1.7 x 10^−5^ or 0.0276), and between *ADAMTS9* rs9831846 and age (P = 0.0019), as shown in Table 5.

**Table 5:** Multivariable logistic regression analysis of SNP-age interaction models for four key SNPs within the *ADAMTS9* gene.

SNP-age interaction model	OR	95% CI	P value^b^
(a) *ADAMTS9* rs73832338			
*ADAMTS9* rs73832338 (CC genotype) with Age ≦ 64 years old^a^	1		
*ADAMTS9* rs73832338 (CC genotype) with Age > 64 years old	0.76	0.45–1.28	0.3014
*ADAMTS9* rs73832338 (TT+TC genotype) with Age ≦ 64 years old	4.90	1.82–13.16	**0.0016**
*ADAMTS9* rs73832338 (TT+TC genotype) with Age > 64 years old	1.30	0.67–2.55	0.4408
(b) *ADAMTS9* rs9985304			
*ADAMTS9* rs9985304 (AA genotype) with Age ≦ 64 years old^a^	1		
*ADAMTS9* rs99853048 (AA genotype) with Age > 64 years old	1.18	0.50–2.75	0.7063
*ADAMTS9* rs9985304 (GG+AG genotype) with Age ≦ 64 years old	6.51	3.08–13.73	**8.8 x 10**^**−7**^
*ADAMTS9* rs9985304 (GG+AG genotype) with Age > 64 years old	2.67	1.35–5.26	**0.0045**
(c) *ADAMTS9* rs4317088			
*ADAMTS9* rs4317088 (CC genotype) with Age ≦ 64 years old^a^	1		
*ADAMTS9* rs4317088 (CC genotype) with Age > 64 years old	0.86	0.40–1.86	0.7098
*ADAMTS9* rs4317088 (TT+TC genotype) with Age ≦ 64 years old	4.86	2.36–9.98	**1.7 x 10**^**−5**^
*ADAMTS9* rs4317088 (TT+TC genotype) with Age > 64 years old	2.05	1.08–3.88	**0.0276**
(d) *ADAMTS9* rs9831846			
*ADAMTS9* rs9831846 (CC genotype) with Age ≦ 64 years old^a^	1		
*ADAMTS9* rs9831846 (CC genotype) with Age > 64 years old	0.71	0.33–1.56	0.3967
*ADAMTS9* rs9831846 (TT+TC genotype) with Age ≦ 64 years old	3.11	1.52–6.39	**0.0019**
*ADAMTS9* rs9831846 (TT+TC genotype) with Age > 64 years old	1.65	0.85–3.21	0.1385

CI = confidence interval, OR = odds ratio.

^a^ Reference.

^b^ Versus reference.

Analysis was obtained after adjustment for covariates including gender and education. P values of < 0.05 are shown in bold.

## Discussion

Our study is the first to date to track down whether the main effects of 96 tag SNPs in three insulin resistance-associated genes, namely the *ADAMTS9*, *GCKR*, and *PPARG* genes, are significantly associated with the risk of cognitive aging independently and/or through SNP-SNP interactions among old Taiwanese individuals. Here, we report for the first time that the *ADAMTS9* gene may play a key role in the modulation of cognitive aging for the elderly in a Taiwanese population. Notably, the significant association of four key SNPs in the *ADAMTS9* gene with MMSE scores persisted after correcting for multiple testing (P < 0.0005). Additionally, our data revealed that SNP-SNP interactions between SNPs of the *ADAMTS9* gene may contribute to the etiology of cognitive aging.

To our knowledge, our results are the first to raise the possibility that four key SNPs (rs73832338, rs9985304, rs4317088, and rs9831846) within the *ADAMTS9* gene may contribute to susceptibility to cognitive aging. Intriguingly, significant associations between these four key SNPs and MMSE scores persisted even after applying Bonferroni correction. According to the aforementioned hypothesis, we speculated that the insulin resistance-relevant genes may be associated with cognitive aging because insulin resistance is considered a risk factor for cognitive aging and AD [3–5]. Not only is insulin a primary contributor for normal brain functioning, but insulin abnormalities also increase the risk for neurodegenerative disorders such as cognitive aging, age-related memory impairment, and AD [6, 7]. Along with our findings, evidence has been reported for an association of the *ADAMTS9* gene with insulin sensitivity, insulin resistance, and T2D [9, 10, 37]. A growing body of evidence further indicates that the ADAMTS-9 protein, which is encoded by the *ADAMTS9* gene, may be involved in physiological conditions and pathological disease states in central nervous system injuries such as ischemic stroke and spinal cord injury [11–13]. In an animal study, Reid et al. suggested that an increased synthesis of the ADAMTS-9 protein was modulated in response to injured neurons in brain tissue after transient middle cerebral artery occlusion [11]. Similarly, in another animal study, Demircan et al. found that ADAMTS-9 expression levels in the mouse spinal cord were upregulated following injury [12]. The *ADAMTS9* rs6795735 SNP was also associated with age-related macular degeneration, which typically occurs in older individuals, in a genome-wide association study [38]. It should be noted that the minor allele frequencies of these four key SNPs vary considerably between different ethnic populations (S4 Table). For example, the A allele frequency of *ADAMTS9* rs9985304 ranges from 42.73% in the present Taiwanese population, 64.29% in British subjects, 47.12% in Japanese subjects, 51.64% in African American subjects, to 52.43% in Han Chinese subjects.

Remarkably, we further inferred the epistatic effects between *ADAMTS9* rs9985304 and *ADAMTS9* rs76346246 on influencing cognitive aging by using the GMDR approach. To our knowledge, there is no prior implication because no other study has been conducted to weigh SNP-SNP interactions between these SNPs. Our analysis implicated that the interaction effect between *ADAMTS9* rs9985304 and *ADAMTS9* rs76346246 on cognitive aging yielded an OR value of 0.11 when we compared individuals carrying the AA genotype of *ADAMTS9* rs9985304 and the C allele of *ADAMTS9* rs76346246 with those carrying the G allele of *ADAMTS9* rs9985304 and the TT genotype of *ADAMTS9* rs76346246. Similarly, this interaction effect yielded an OR value of 0.37 when we compared individuals carrying the AA genotype of *ADAMTS9* rs9985304 and the G allele of *ADAMTS9* rs76346246 with those carrying the G allele of *ADAMTS9* rs9985304 and the TT genotype of *ADAMTS9* rs76346246. It also yielded an OR value of 0.2 when we compared individuals carrying the AA genotype of *ADAMTS9* rs9985304 and the TT genotype of *ADAMTS9* rs76346246 with those carrying the G allele of *ADAMTS9* rs9985304 and the TT genotype of *ADAMTS9* rs76346246. The biological effects of synergy between *ADAMTS9* rs9985304 and *ADAMTS9* rs76346246 on cognitive aging remain to be elucidated. It should be pointed out that the C allele frequency of *ADAMTS9* rs76346246 differs noticeably between various ethnic populations, ranging from 7.02% in the present Taiwanese population, 0.00% in British subjects, 7.69% in Japanese subjects, 0.82% in African American subjects, to 9.71% in Han Chinese subjects as shown in public data from the 1000 Genomes Project (S4 Table).

Among the strengths of our study is having access to a large Taiwanese cohort such as the Taiwan Biobank [27–30] to explore the role of insulin resistance-associated genes in relation to cognitive aging. On the other hand, a main weakness is that our findings require prospective clinical trials to gauge whether the present observations are sustained in varied ethnic groups [39–41]. While all study subjects self-reported as being of Taiwanese Han Chinese ancestry, this study may also fail to detect potential population stratification, because it is difficult to collect a large sample with a homogeneous genetic background due to logistical and ethical reasons [29]. Further studies with other ethnic populations are envisioned to build up a thorough evaluation of the association and interactions of the investigated variants with cognitive aging [42–44].

## Conclusions

In conclusion, we conducted an extensive analysis of the association of cognitive aging with insulin resistance-associated genes, namely the *ADAMTS9*, *GCKR*, and *PPARG* genes in older Taiwanese adults. We also examined the influence of SNP-SNP interactions in these genes on cognitive aging. Overall, the results from the current study, if replicated in independent and statistically well-powered studies, suggest the impacts of the *ADAMTS9* gene on the prevalence of cognitive aging independently and/or through complex SNP-SNP interactions. Independent replication studies with a much larger number of participants will likely demonstrate further insights into the role of the *ADAMTS9* gene pinpointed in this study.

## Supporting information

S1 Table Genotyping results for 141 SNPs in three insulin resistance-related genes including the *ADAMTS9*, *GCKR*, and *PPARG* genes. (DOC)

S2 Table 96 tag SNPs in three insulin resistance-related genes including the *ADAMTS9*, *GCKR*, and *PPARG* genes. (DOC)

S3 Table Linear regression models of associations between the MMSE scores and 96 tag SNPs in three insulin resistance-related genes including the *ADAMTS9*, *GCKR*, and *PPARG* genes. (DOC)

S4 Table MAF in various ethnic populations for five tag SNPs in the *ADAMTS9* gene. (DOC)

## References

[1] Wang KC, Woung LC, Tsai MT, Liu CC, Su YH, Li CY. Risk of Alzheimer's disease in relation to diabetes: a population-based cohort study. Neuroepidemiology. 2012; 38: 237–44. 10.1159/000337428 22572745

[2] Vagelatos NT, Eslick GD. Type 2 diabetes as a risk factor for Alzheimer's disease: the confounders, interactions, and neuropathology associated with this relationship. Epidemiol Rev. 2013; 35: 152–60. 10.1093/epirev/mxs012 23314404

[3] Hao K, Di Narzo AF, Ho L, Luo W, Li S, Chen R, et al Shared genetic etiology underlying Alzheimer's disease and type 2 diabetes. Mol Aspects Med. 2015; 43–44: 66–76. 10.1016/j.mam.2015.06.006 26116273 PMC6021176

[4] Gao L, Cui Z, Shen L, Ji HF. Shared Genetic Etiology between Type 2 Diabetes and Alzheimer's Disease Identified by Bioinformatics Analysis. J Alzheimers Dis. 2015; 50: 13–7. 10.3233/JAD-150580 26639962

[5] Watson GS, Craft S. The role of insulin resistance in the pathogenesis of Alzheimer's disease: implications for treatment. CNS Drugs. 2003; 17: 27–45. 12467491 10.2165/00023210-200317010-00003

[6] Watson GS, Craft S. Insulin resistance, inflammation, and cognition in Alzheimer's Disease: lessons for multiple sclerosis. J Neurol Sci. 2006; 245: 21–33. 10.1016/j.jns.2005.08.017 16631207

[7] Craft S. Insulin resistance and Alzheimer's disease pathogenesis: potential mechanisms and implications for treatment. Curr Alzheimer Res. 2007; 4: 147–52. 17430239 10.2174/156720507780362137

[8] Neumann KF, Rojo L, Navarrete LP, Farías G, Reyes P, Maccioni RB. Insulin resistance and Alzheimer's disease: molecular links & clinical implications. Curr Alzheimer Res. 2008; 5: 438–47. 18855585 10.2174/156720508785908919

[9] Boesgaard TW, Gjesing AP, Grarup N, Rutanen J, Jansson PA, Hribal ML, et al Variant near ADAMTS9 known to associate with type 2 diabetes is related to insulin resistance in offspring of type 2 diabetes patients—EUGENE2 study. PLoS One. 2009; 4: e7236 10.1371/journal.pone.0007236 19789630 PMC2747270

[10] Trombetta M, Bonetti S, Boselli ML, Miccoli R, Trabetti E, Malerba G, et al PPARG2 Pro12Ala and ADAMTS9 rs4607103 as "insulin resistance loci" and "insulin secretion loci" in Italian individuals. The GENFIEV study and the Verona Newly Diagnosed Type 2 Diabetes Study (VNDS) 4. Acta Diabetol. 2013; 50: 401–8. 10.1007/s00592-012-0443-9 23161442

[11] Reid MJ, Cross AK, Haddock G, Allan SM, Stock CJ, Woodroofe MN, et al ADAMTS-9 expression is up-regulated following transient middle cerebral artery occlusion (tMCAo) in the rat. Neurosci Lett. 2009; 452: 252–257. 10.1016/j.neulet.2009.01.058 19348733

[12] Demircan K, Yonezawa T, Takigawa T, Topcu V, Erdogan S, Ucar F, et al ADAMTS1, ADAMTS5, ADAMTS9 and aggrecanase-generated proteoglycan fragments are induced following spinal cord injury in mouse. Neurosci Lett. 2013, 544:25–30. 10.1016/j.neulet.2013.02.064 23562508

[13] Lemarchant S, Pruvost M, Montaner J, Emery E, Vivien D, Kanninen K, et al ADAMTS proteoglycanases in the physiological and pathological central nervous system. J Neuroinflammation. 2013; 10: 133 10.1186/1742-2094-10-133 24176075 PMC4228433

[14] Sparsø T, Andersen G, Nielsen T, Burgdorf KS, Gjesing AP, Nielsen AL, et al The GCKR rs780094 polymorphism is associated with elevated fasting serum triacylglycerol, reduced fasting and OGTT-related insulinaemia, and reduced risk of type 2 diabetes. Diabetologia. 2008; 51: 70–5. 10.1007/s00125-007-0865-z 18008060

[15] Roncero I, Alvarez E, Chowen JA, Sanz C, Rábano A, Vázquez P, et al Expression of glucose transporter isoform GLUT-2 and glucokinase genes in human brain. J Neurochem. 2004; 88: 1203–10. 15009676 10.1046/j.1471-4159.2003.02269.x

[16] Roncero I, Sanz C, Alvarez E, Vázquez P, Barrio PA, Blázquez E. Glucokinase and glucokinase regulatory proteins are functionally coexpressed before birth in the rat brain. J Neuroendocrinol. 2009; 21: 973–81. 10.1111/j.1365-2826.2009.01919.x 19807849

[17] Onuma H, Tabara Y, Kawamoto R, Shimizu I, Kawamura R, Takata Y, et al The GCKR rs780094 polymorphism is associated with susceptibility of type 2 diabetes, reduced fasting plasma glucose levels, increased triglycerides levels and lower HOMA-IR in Japanese population. J Hum Genet. 2010; 55: 600–4. 10.1038/jhg.2010.75 20574426

[18] Køster B, Fenger M, Poulsen P, Vaag A, Bentzen J. Novel polymorphisms in the GCKR gene and their influence on glucose and insulin levels in a Danish twin population. Diabet Med. 2005; 22: 1677–82. 10.1111/j.1464-5491.2005.01700.x 16401311

[19] Vidal-Puig AJ, Considine RV, Jimenez-Liñan M, Werman A, Pories WJ, Caro JF, et al Peroxisome proliferator-activated receptor gene expression in human tissues. Effects of obesity, weight loss, and regulation by insulin and glucocorticoids. J Clin Invest. 1997; 99: 2416–22. 10.1172/JCI119424 9153284 PMC508081

[20] Hsiao TJ, Lin E. The Pro12Ala polymorphism in the peroxisome proliferator-activated receptor gamma (PPARG) gene in relation to obesity and metabolic phenotypes in a Taiwanese population. Endocrine. 2015; 48: 786–93. 10.1007/s12020-014-0407-7 25182148

[21] Deeb SS, Fajas L, Nemoto M, Pihlajamäki J, Mykkänen L, Kuusisto J, et al A Pro12Ala substitution in PPARgamma2 associated with decreased receptor activity, lower body mass index and improved insulin sensitivity. Nat Genet. 1998; 20: 284–7. 10.1038/3099 9806549

[22] González Sánchez JL, Serrano Ríos M, Fernández Perez C, Laakso M, Martínez Larrad MT. Effect of the Pro12Ala polymorphism of the peroxisome proliferator-activated receptor gamma-2 gene on adiposity, insulin sensitivity and lipid profile in the Spanish population. Eur J Endocrinol. 2002; 147: 495–501. 12370112 10.1530/eje.0.1470495

[23] Bhatt SP, Misra A, Sharma M, Luthra K, Guleria R, Pandey RM, et al Ala/Ala genotype of Pro12Ala polymorphism in the peroxisome proliferator-activated receptor-γ2 gene is associated with obesity and insulin resistance in Asian Indians. Diabetes Technol Ther. 2012; 14: 828–34. 10.1089/dia.2011.0277 22694222 PMC3429329

[24] West NA, Haan MN, Morgenstern H. The PPAR-gamma Pro12Ala polymorphism and risk of cognitive impairment in a longitudinal study. Neurobiol Aging. 2010; 31: 741–6. 10.1016/j.neurobiolaging.2008.06.005 18639367 PMC3099450

[25] Black MH, Wu J, Takayanagi M, Wang N, Taylor KD, Haritunians T, et al Variation in PPARG is associated with longitudinal change in insulin resistance in Mexican Americans at risk for type 2 diabetes. J Clin Endocrinol Metab. 2015; 100: 1187–95. 10.1210/jc.2014-3246 25584717 PMC4333029

[26] Watson GS, Cholerton BA, Reger MA, Baker LD, Plymate SR, Asthana S, et al Preserved cognition in patients with early Alzheimer disease and amnestic mild cognitive impairment during treatment with rosiglitazone: a preliminary study. Am J Geriatr Psychiatry. 2005; 13: 950–8. 10.1176/appi.ajgp.13.11.950 16286438

[27] Fan CT, Lin JC, Lee CH. Taiwan Biobank: a project aiming to aid Taiwan's transition into a biomedical island. Pharmacogenomics. 2008; 9: 235–46. 10.2217/14622416.9.2.235 18370851

[28] Lin E, Kuo PH, Liu YL, Yang AC, Kao CF, Tsai SJ. Association and interaction of APOA5, BUD13, CETP, LIPA and health-related behavior with metabolic syndrome in a Taiwanese population. Sci Rep. 2016; 6: 36830 10.1038/srep36830 27827461 PMC5101796

[29] Chen CH, Yang JH, Chiang CW, Hsiung CN, Wu PE, Chang LC, et al Population structure of Han Chinese in the modern Taiwanese population based on 10,000 participants in the Taiwan Biobank project. Hum Mol Genet. 2016 [Epub ahead of print]. 10.1093/hmg/ddw346 PMC6078601 27798100

[30] Lin E, Tsai SJ, Kuo PH, Liu YL, Yang AC, Kao CF, Yang CH. The rs1277306 variant of the REST gene confers susceptibility to cognitive aging in the elderly in a Taiwanese population. Dement Geriatr Cogn Disord. 2016; Forthcoming. 10.1159/000455833 28142142

[31] Sheehan B. Assessment scales in dementia. Ther Adv Neurol Disord. 2012; 5:349–58. 10.1177/1756285612455733 23139705 PMC3487532

[32] Liou YJ, Bai YM, Lin E, Chen JY, Chen TT, Hong CJ, et al Gene-gene interactions of the INSIG1 and INSIG2 in metabolic syndrome in schizophrenic patients treated with atypical antipsychotics. Pharmacogenomics J. 2012; 12: 54–61 10.1038/tpj.2010.74 20877301

[33] Hsiao TJ, Lin E. The ENPP1 K121Q polymorphism is associated with type 2 diabetes and related metabolic phenotypes in a Taiwanese population. Mol Cell Endocrinol. 2016; 433: 20–25. 10.1016/j.mce.2016.05.020 27238374

[34] Purcell S, Neale B, Todd-Brown K, Thomas L, Ferreira MA, Bender D, et al PLINK: a tool set for whole-genome association and population-based linkage analyses. Am J Hum Genet. 2007; 81:559–75. 10.1086/519795 17701901 PMC1950838

[35] Hsiao TJ, Hwang Y, Liu CH, Chang HM, Lin E. Association of the C825T polymorphism in the GNB3 gene with obesity and metabolic phenotypes in a Taiwanese population. Genes Nutr. 2013; 8: 137–44. 10.1007/s12263-012-0304-8 22791279 PMC3534990

[36] Lou XY, Chen GB, Yan L, Ma JZ, Zhu J, Elston RC, et al A generalized combinatorial approach for detecting gene-by-gene and gene-by-environment interactions with application to nicotine dependence. Am J Hum Genet. 2007; 80: 1125–1137. 10.1086/518312 17503330 PMC1867100

[37] Zeggini E, Scott LJ, Saxena R, Voight BF, Marchini JL, Hu T, et al Meta-analysis of genome-wide association data and large-scale replication identifies additional susceptibility loci for type 2 diabetes. Nat Genet. 2008; 40: 638–45. 10.1038/ng.120 18372903 PMC2672416

[38] Fritsche LG, Chen W, Schu M, Yaspan BL, Yu Y, Thorleifsson G, et al Seven new loci associated with age-related macular degeneration. Nat Genet. 2013; 45: 433–9, 439e1-2. 10.1038/ng.2578 23455636 PMC3739472

[39] Hsiao TJ, Hwang Y, Chang HM, Lin E. Association of the rs6235 variant in the proprotein convertase subtilisin/kexin type 1 (PCSK1) gene with obesity and related traits in a Taiwanese population. Gene. 2014; 533: 32–7. 10.1016/j.gene.2013.10.016 24140494

[40] Hsiao TJ, Lin E. A common rs7903146 variant of the transcription factor 7 like 2 gene is associated with type 2 diabetes mellitus and fasting glucose in a Taiwanese population. Diabetes Metab. (in press) 10.1016/j.diabet.2016.05.003 27286737

[41] Hsiao TJ, Lin E. Evaluation of the glutamine 27 glutamic acid polymorphism in the adrenoceptor beta 2 surface gene on obesity and metabolic phenotypes in Taiwan. J Investig Med. 2014; 62: 310–5. 10.2310/JIM.0000000000000030 24322328

[42] Lane HY, Tsai GE, Lin E. Assessing gene-gene interactions in pharmacogenomics. Mol Diagn Ther. 2012; 16: 15–27. 10.2165/11597270-000000000-00000 22352452

[43] Hsiao TJ, Lin E. Association of a common rs9939609 variant in the fat mass and obesity-associated (FTO) gene with obesity and metabolic phenotypes in a Taiwanese population: a replication study. J Genet. 2016; 95: 595–601. 27659330 10.1007/s12041-016-0671-9

[44] Hsiao TJ, Lin E. A validation study of adiponectin rs266729 gene variant with type 2 diabetes, obesity, and metabolic phenotypes in a Taiwanese population. Biochem Genet. 2016; 54: 830–841. 10.1007/s10528-016-9760-y 27388775

