# Supplementary material for: The ADAMTS9 gene is associated with cognitive aging in the elderly in a Taiwanese population
Source: PLoS One. 2017 Feb 22;12(2):e0172440. doi: 10.1371/journal.pone.0172440 (PMC5321460; doi:10.1371/journal.pone.0172440)
Supplement: S1 Table — (DOC) [file pone.0172440.s001.doc]

**S1 Table.** Genotyping results for 141 SNPs in three insulin resistance-related genes including the *ADAMTS9*, *GCKR*, and *PPARG* genes.

| Gene | CHR | SNP | A1 | A2 | MAF | P (HWE) | Genotyping call rate |
| --- | --- | --- | --- | --- | --- | --- | --- |
| *ADAMTS9* | 3 | rs67920064 | A | G | 0.393 | 0.970 | 1.000 |
|  |  | rs10470705 | G | A | 0.343 | 0.935 | 0.998 |
|  |  | rs13095481 | C | T | 0.429 | 0.628 | 0.997 |
|  |  | rs13093187 | C | T | 0.406 | 0.362 | 0.995 |
|  |  | rs7625847 | G | A | 0.306 | 1.000 | 1.000 |
|  |  | rs78246822 | A | G | 0.051 | **< 0.001** | 1.000 |
|  |  | rs17070941 | T | C | 0.296 | 0.174 | 0.995 |
|  |  | rs9866261 | A | G | 0.475 | 0.057 | 0.998 |
|  |  | rs17070967 | C | T | 0.077 | 0.070 | 0.998 |
|  |  | rs1036919 | T | C | 0.401 | 0.057 | 0.997 |
|  |  | rs78826033 | G | T | 0.195 | 0.449 | 0.998 |
|  |  | rs894744 | G | A | 0.308 | 0.607 | 0.995 |
|  |  | rs11130971 | G | A | 0.407 | 0.112 | 0.998 |
|  |  | rs77263040 | C | T | 0.144 | **0.017** | 1.000 |
|  |  | rs9311896 | T | C | 0.300 | 0.728 | 0.998 |
|  |  | rs17071023 | G | C | 0.103 | 0.167 | 0.997 |
|  |  | rs34634062 | T | G | 0.249 | **< 0.001** | 0.980 |
|  |  | rs12629732 | G | A | 0.209 | **0.009** | 0.998 |
|  |  | rs12637910 | T | C | 0.282 | 0.162 | 1.000 |
|  |  | rs6782835 | C | T | 0.351 | 0.718 | 0.998 |
|  |  | rs1561988 | A | G | 0.496 | 0.715 | 1.000 |
|  |  | rs1036920 | A | G | 0.229 | 0.098 | 0.995 |
|  |  | rs1014640 | T | C | 0.441 | 0.111 | 0.998 |
|  |  | rs17071042 | C | A | 0.076 | 0.897 | 1.000 |
|  |  | rs76823504 | C | A | 0.078 | 1.000 | 0.994 |
|  |  | rs4642117 | T | C | 0.463 | 0.591 | **0.882** |
|  |  | rs7625654 | A | G | 0.141 | **0.049** | 1.000 |
|  |  | rs17071079 | C | T | 0.313 | 0.641 | 0.998 |
|  |  | rs17071083 | T | C | 0.259 | 0.962 | 1.000 |
|  |  | rs79501236 | A | G | 0.233 | 0.081 | 0.995 |
|  |  | rs17726803 | T | C | 0.335 | 0.870 | 0.998 |
|  |  | rs4431100 | C | T | 0.484 | 0.714 | 0.998 |
|  |  | rs73832332 | T | C | 0.236 | 0.223 | 0.998 |
|  |  | rs9820942 | A | G | 0.243 | 0.457 | 0.997 |
|  |  | rs3935273 | T | C | 0.218 | 0.592 | 0.998 |
|  |  | rs13318141 | T | C | 0.315 | 0.099 | 0.997 |
|  |  | rs76346246 | C | T | 0.070 | 0.574 | 0.998 |
|  |  | rs7648540 | C | A | 0.245 | 0.693 | 1.000 |
|  |  | rs75839462 | C | T | 0.069 | 0.887 | 0.998 |
|  |  | rs79126323 | C | T | 0.282 | **0.038** | 1.000 |
|  |  | rs9311899 | C | T | 0.430 | 0.297 | 0.998 |
|  |  | rs7614362 | C | A | 0.170 | 0.746 | 0.998 |
|  |  | rs117355594 | T | C | 0.069 | 0.887 | 1.000 |
|  |  | rs79601438 | G | A | 0.262 | 0.449 | 0.997 |
|  |  | rs4393895 | T | A | 0.400 | **0.010** | 0.991 |
|  |  | rs77320716 | C | T | 0.264 | 0.348 | 1.000 |
|  |  | rs77076497 | C | A | 0.048 | 0.228 | 1.000 |
|  |  | rs4405909 | A | G | 0.338 | 0.541 | 1.000 |
|  |  | rs60073432 | A | G | 0.251 | 0.698 | 0.998 |
|  |  | rs73832338 | T | C | 0.207 | 0.697 | 0.998 |
|  |  | rs9864390 | C | G | 0.311 | 0.966 | 1.000 |
|  |  | rs9985304 | A | G | 0.427 | 0.314 | 0.998 |
|  |  | rs6784609 | C | T | 0.487 | 0.154 | 1.000 |
|  |  | rs4688490 | A | G | 0.431 | 0.263 | 0.994 |
|  |  | rs4317088 | C | T | 0.490 | 0.079 | 0.997 |
|  |  | rs4589926 | T | C | 0.488 | **0.040** | 0.997 |
|  |  | rs6802863 | T | C | 0.436 | 0.169 | 0.997 |
|  |  | rs4340697 | T | C | 0.243 | 0.843 | 1.000 |
|  |  | rs7636925 | A | G | 0.438 | 0.504 | 0.998 |
|  |  | rs6445420 | C | T | 0.083 | 0.335 | 0.986 |
|  |  | rs9835360 | T | G | 0.487 | 0.854 | 0.992 |
|  |  | rs12053983 | A | G | 0.206 | 0.867 | 1.000 |
|  |  | rs9831846 | C | T | 0.484 | 0.913 | 1.000 |
|  |  | rs9832057 | T | C | 0.299 | **< 0.001** | 0.992 |
|  |  | rs7632802 | T | C | 0.307 | 0.213 | 0.998 |
|  |  | rs76042002 | T | C | 0.050 | 0.558 | 1.000 |
|  |  | rs11916325 | A | C | 0.176 | 0.285 | 0.998 |
|  |  | rs9866907 | A | C | 0.116 | 1.000 | 1.000 |
|  |  | rs9868005 | A | C | 0.157 | 0.890 | 1.000 |
|  |  | rs13095235 | A | C | 0.037 | **0.032** | 0.976 |
|  |  | rs9861153 | C | T | 0.261 | 0.538 | 0.997 |
|  |  | rs4371513 | A | G | 0.419 | 0.910 | 0.994 |
|  |  | rs4605539 | T | C | 0.073 | 0.786 | 1.000 |
|  |  | rs9851598 | A | G | 0.289 | 0.790 | 0.998 |
|  |  | rs11130975 | A | G | 0.455 | 0.883 | 0.997 |
|  |  | rs6768305 | G | C | 0.070 | 1.000 | 1.000 |
|  |  | rs7615771 | T | C | 0.070 | 0.889 | 1.000 |
|  |  | rs9855230 | G | A | 0.122 | 0.267 | 1.000 |
|  |  | rs7646362 | A | G | 0.046 | 0.679 | 1.000 |
|  |  | rs11429228 | N | C | 0.124 | 0.273 | 0.997 |
|  |  | rs75581931 | A | G | 0.224 | 0.958 | 0.994 |
|  |  | rs80118777 | G | T | 0.150 | 0.281 | 0.995 |
|  |  | rs13320442 | G | A | 0.403 | 0.448 | 0.998 |
|  |  | rs79062861 | T | C | 0.085 | 0.551 | 0.967 |
|  |  | rs7623988 | G | C | 0.313 | 0.899 | 0.994 |
|  |  | rs9836710 | G | A | 0.117 | 0.723 | 1.000 |
|  |  | rs11921149 | A | G | 0.365 | 0.875 | 1.000 |
|  |  | rs7619937 | A | G | 0.496 | 0.342 | 0.997 |
|  |  | rs4579012 | T | C | 0.428 | 0.205 | 1.000 |
|  |  | rs7642530 | A | G | 0.426 | 0.501 | 0.997 |
|  |  | rs6793277 | G | T | 0.207 | 0.738 | 0.997 |
|  |  | rs4522762 | C | T | 0.117 | 0.481 | 0.997 |
|  |  | rs13434166 | G | A | 0.140 | 0.198 | 0.998 |
|  |  | rs80303166 | G | A | 0.098 | 1.000 | 0.998 |
|  |  | rs4566532 | G | T | 0.397 | 0.127 | 1.000 |
|  |  | rs4505693 | G | T | 0.396 | 0.118 | 1.000 |
|  |  | rs4637287 | G | C | 0.183 | 0.855 | 0.998 |
|  |  | rs58650552 | T | C | 0.186 | 0.904 | 1.000 |
|  |  | rs6776363 | C | G | 0.347 | 0.572 | 1.000 |
|  |  | rs12492549 | G | C | 0.447 | 0.941 | 1.000 |
|  |  | rs73124286 | T | G | 0.408 | 1.000 | 1.000 |
| *GCKR* | 2 | rs1260326 | T | C | 0.485 | 0.942 | 0.997 |
|  |  | rs4425043 | A | G | 0.143 | 0.264 | 0.998 |
|  |  | rs780094 | T | C | 0.469 | 0.769 | 1.000 |
|  |  | rs780093 | T | C | 0.472 | 0.769 | 0.998 |
|  |  | rs780092 | G | A | 0.373 | 0.242 | 0.998 |
|  |  | rs8179252 | C | A | 0.153 | 0.232 | 1.000 |
| *PPARG* | 3 | rs73021485 | T | G | 0.385 | 0.275 | 0.989 |
|  |  | rs6782178 | T | C | 0.046 | 0.833 | 1.000 |
|  |  | rs2960422 | A | G | 0.446 | 0.301 | 1.000 |
|  |  | rs73023314 | A | C | 0.091 | 0.098 | 0.998 |
|  |  | rs12636461 | A | G | 0.499 | 0.401 | 0.998 |
|  |  | rs11710969 | A | G | 0.500 | 0.443 | 0.998 |
|  |  | rs10510411 | A | G | 0.358 | 0.283 | 0.994 |
|  |  | rs60290266 | A | G | 0.359 | 0.302 | 0.998 |
|  |  | rs4684101 | C | T | 0.359 | 0.340 | 0.997 |
|  |  | rs75512179 | C | T | 0.083 | 0.546 | 0.998 |
|  |  | rs12490265 | A | G | 0.097 | 0.403 | 0.998 |
|  |  | rs10510418 | C | A | 0.158 | 0.491 | 0.994 |
|  |  | rs7649970 | T | C | 0.039 | 0.807 | 0.997 |
|  |  | rs1801282 | G | C | 0.039 | 0.806 | 1.000 |
|  |  | rs4135247 | A | G | 0.458 | 0.461 | 1.000 |
|  |  | rs17817276 | G | A | 0.099 | 0.608 | 1.000 |
|  |  | rs13306745 | T | G | 0.073 | 0.417 | 1.000 |
|  |  | rs2972162 | T | C | 0.409 | 0.597 | 0.995 |
|  |  | rs4135268 | G | C | 0.053 | 0.718 | 0.995 |
|  |  | rs4135275 | G | A | 0.499 | 0.608 | 0.997 |
|  |  | rs4135283 | A | C | 0.312 | 0.671 | 1.000 |
|  |  | rs709149 | A | G | 0.411 | 0.474 | 0.998 |
|  |  | rs117209672 | A | C | 0.088 | 1.000 | 0.997 |
|  |  | rs1152001 | G | A | 0.098 | 0.756 | 0.998 |
|  |  | rs3856806 | T | C | 0.246 | 0.325 | 1.000 |
|  |  | rs1152003 | C | G | 0.480 | 0.798 | 0.998 |
|  |  | rs9833097 | A | G | 0.001 | 1.000 | 0.998 |
|  |  | rs17819328 | T | G | 0.069 | 0.197 | 1.000 |
|  |  | rs78287138 | T | C | 0.142 | 1.000 | 1.000 |
|  |  | rs9809905 | G | T | 0.104 | 0.097 | 1.000 |
|  |  | rs4256108 | A | G | 0.069 | 1.000 | 1.000 |
|  |  | rs11917039 | C | G | 0.052 | 1.000 | 0.997 |
|  |  | rs17036788 | C | T | 0.051 | 0.851 | 0.998 |
|  |  | rs709166 | A | G | 0.086 | 0.414 | 0.998 |

A1 = minor allele, A2 = major allele, Chr = chromosome, HWE = Hardy–Weinberg equilibrium, MAF = minor allele frequency, MMSE = Mini-Mental State Examination.

P values of < 0.05 are shown in bold.

Genotyping call rate values of < 0.95 are shown in bold.
