# Supplementary material for: The ADAMTS9 gene is associated with cognitive aging in the elderly in a Taiwanese population
Source: PLoS One. 2017 Feb 22;12(2):e0172440. doi: 10.1371/journal.pone.0172440 (PMC5321460; doi:10.1371/journal.pone.0172440)
Supplement: S2 Table — (DOC) [file pone.0172440.s002.doc]

**S2 Table.** 96 tag SNPs in three insulin resistance-related genes including the *ADAMTS9*, *GCKR*, and *PPARG* genes.

| Gene | CHR | SNP | With other SNPs in strong LD (r^2^ > 0.8) |
| --- | --- | --- | --- |
| *ADAMTS9* | 3 | rs67920064 |  |
|  |  | rs10470705 |  |
|  |  | rs13095481 |  |
|  |  | rs13093187 | rs1036919, rs11130971 |
|  |  | rs7625847 | rs894744, rs9311896 |
|  |  | rs17070941 |  |
|  |  | rs9866261 |  |
|  |  | rs17070967 |  |
|  |  | rs78826033 |  |
|  |  | rs17071023 |  |
|  |  | rs12637910 |  |
|  |  | rs6782835 |  |
|  |  | rs1561988 |  |
|  |  | rs1036920 | rs79501236, rs73832332 |
|  |  | rs1014640 |  |
|  |  | rs17071042 | rs76823504 |
|  |  | rs17071079 |  |
|  |  | rs17071083 |  |
|  |  | rs17726803 |  |
|  |  | rs4431100 |  |
|  |  | rs9820942 | rs7648540 |
|  |  | rs3935273 |  |
|  |  | rs13318141 |  |
|  |  | rs76346246 | rs75839462, rs117355594 |
|  |  | rs9311899 |  |
|  |  | rs7614362 |  |
|  |  | rs79601438 | rs77320716 |
|  |  | rs77076497 |  |
|  |  | rs4405909 |  |
|  |  | rs60073432 | rs4340697 |
|  |  | rs73832338 | rs12053983 |
|  |  | rs9864390 |  |
|  |  | rs9985304 | rs4688490, rs6802863, rs7636925 |
|  |  | rs4317088 | rs6784609, rs6802863, rs9835360 |
|  |  | rs6445420 |  |
|  |  | rs9831846 |  |
|  |  | rs7632802 |  |
|  |  | rs76042002 |  |
|  |  | rs11916325 |  |
|  |  | rs9866907 |  |
|  |  | rs9868005 |  |
|  |  | rs9861153 | rs9851598 |
|  |  | rs4371513 | rs11130975 |
|  |  | rs4605539 |  |
|  |  | rs6768305 | rs7615771 |
|  |  | rs9855230 |  |
|  |  | rs7646362 |  |
|  |  | rs11429228 |  |
|  |  | rs75581931 |  |
|  |  | rs80118777 |  |
|  |  | rs13320442 |  |
|  |  | rs79062861 |  |
|  |  | rs7623988 |  |
|  |  | rs9836710 |  |
|  |  | rs11921149 |  |
|  |  | rs7619937 |  |
|  |  | rs4579012 |  |
|  |  | rs7642530 |  |
|  |  | rs6793277 |  |
|  |  | rs4522762 |  |
|  |  | rs13434166 |  |
|  |  | rs80303166 |  |
|  |  | rs4566532 | rs4505693 |
|  |  | rs4637287 | rs58650552 |
|  |  | rs6776363 |  |
|  |  | rs12492549 |  |
|  |  | rs73124286 |  |
| *GCKR* | 2 | rs1260326 | rs780094, rs780093 |
|  |  | rs4425043 | rs8179252 |
|  |  | rs780092 |  |
| *PPARG* | 3 | rs73021485 |  |
|  |  | rs6782178 |  |
|  |  | rs2960422 | rs11710969 |
|  |  | rs73023314 | rs75512179 |
|  |  | rs12636461 | rs11710969 |
|  |  | rs10510411 | rs60290266, rs4684101 |
|  |  | rs12490265 | rs75512179, rs17817276 |
|  |  | rs10510418 |  |
|  |  | rs1801282 | rs7649970 |
|  |  | rs4135247 |  |
|  |  | rs13306745 |  |
|  |  | rs2972162 | rs709149 |
|  |  | rs4135268 |  |
|  |  | rs4135275 |  |
|  |  | rs4135283 |  |
|  |  | rs117209672 |  |
|  |  | rs1152001 |  |
|  |  | rs3856806 |  |
|  |  | rs1152003 |  |
|  |  | rs9833097 |  |
|  |  | rs17819328 |  |
|  |  | rs78287138 |  |
|  |  | rs9809905 |  |
|  |  | rs4256108 |  |
|  |  | rs11917039 | rs17036788 |
|  |  | rs709166 |  |

Chr = chromosome, LD = linkage disequilibrium.
