# Supplementary material for: The ADAMTS9 gene is associated with cognitive aging in the elderly in a Taiwanese population
Source: PLoS One. 2017 Feb 22;12(2):e0172440. doi: 10.1371/journal.pone.0172440 (PMC5321460; doi:10.1371/journal.pone.0172440)
Supplement: S3 Table — (DOC) [file pone.0172440.s003.doc]

**S3 Table.** Linear regression models of associations between the MMSE scores and 96 tag SNPs in three insulin resistance-related genes including the *ADAMTS9*, *GCKR*, and *PPARG* genes.

| Gene | CHR | SNP | A1 | A2 | P (additive) | P (recessive) | P (dominant) |
| --- | --- | --- | --- | --- | --- | --- | --- |
| *ADAMTS9* | 3 | rs67920064 | A | G | 0.8385 | 0.9276 | 0.4744 |
|  |  | rs10470705 | G | A | 0.2668 | 0.2511 | 0.7143 |
|  |  | rs13095481 | C | T | 0.8168 | 0.4292 | 0.3427 |
|  |  | rs13093187 | C | T | 0.9729 | 0.8541 | 0.7170 |
|  |  | rs7625847 | G | A | 0.5236 | 0.5522 | 0.6702 |
|  |  | rs17070941 | T | C | 0.1926 | 0.1722 | 0.6714 |
|  |  | rs9866261 | A | G | 0.3167 | 0.3230 | 0.5217 |
|  |  | rs17070967 | C | T | 0.9574 | 0.9716 | **0.0264** |
|  |  | rs78826033 | G | T | 0.2563 | 0.2845 | 0.3754 |
|  |  | rs17071023 | G | C | 0.8891 | 0.8951 | 0.8160 |
|  |  | rs12637910 | T | C | 0.9361 | 0.7467 | 0.3289 |
|  |  | rs6782835 | C | T | 0.3904 | 0.6554 | 0.1787 |
|  |  | rs1561988 | A | G | **0.0138** | **0.0054** | 0.2316 |
|  |  | rs1036920 | A | G | 0.2571 | 0.2117 | 0.9583 |
|  |  | rs1014640 | T | C | **0.0034** | **0.0010** | 0.2344 |
|  |  | rs17071042 | C | A | 0.5527 | 0.5170 | **0.0448** |
|  |  | rs17071079 | C | T | 0.2471 | 0.3540 | 0.3337 |
|  |  | rs17071083 | T | C | 0.1582 | 0.1558 | 0.5529 |
|  |  | rs17726803 | T | C | 0.1743 | 0.1990 | 0.3940 |
|  |  | rs4431100 | C | T | 0.7101 | 0.5955 | 0.8036 |
|  |  | rs9820942 | A | G | 0.5985 | 0.8952 | 0.0689 |
|  |  | rs3935273 | T | C | 0.3097 | 0.4918 | **0.0185** |
|  |  | rs13318141 | T | C | 0.8672 | 0.9536 | 0.5255 |
|  |  | rs76346246 | C | T | 0.9818 | 0.9499 | **0.0047** |
|  |  | rs9311899 | C | T | 0.8573 | 0.8618 | 0.5408 |
|  |  | rs7614362 | C | A | 0.1218 | 0.1289 | 0.4471 |
|  |  | rs79601438 | G | A | 0.7344 | 0.3655 | 0.0617 |
|  |  | rs77076497 | C | A | 0.2830 | 0.2848 | 0.6799 |
|  |  | rs4405909 | A | G | 0.0966 | 0.1159 | 0.3142 |
|  |  | rs60073432 | A | G | 0.2344 | 0.4860 | **0.0184** |
|  |  | rs73832338 | T | C | **0.0012** | **0.0042** | **0.0002** |
|  |  | rs9864390 | C | G | 0.3791 | 0.4763 | 0.3902 |
|  |  | rs9985304 | A | G | **1.4 x 10^-5^** | **4.5 x 10^-5^** | **0.0040** |
|  |  | rs4317088 | C | T | **1.5 x 10^-6^** | **3.7 x 10^-5^** | **0.0002** |
|  |  | rs6445420 | C | T | **0.0221** | **0.0232** | 0.2595 |
|  |  | rs9831846 | C | T | **2.2 x 10^-5^** | **0.0002** | **0.0013** |
|  |  | rs7632802 | T | C | 0.9535 | 0.7557 | 0.5068 |
|  |  | rs76042002 | T | C | 0.1256 | 0.1294 | 0.3189 |
|  |  | rs11916325 | A | C | 0.8161 | 0.7831 | 0.7624 |
|  |  | rs9866907 | A | C | 0.1251 | 0.1258 | 0.6635 |
|  |  | rs9868005 | A | C | 0.3487 | 0.3343 | 0.9940 |
|  |  | rs9861153 | C | T | 0.3032 | 0.3495 | 0.4071 |
|  |  | rs4371513 | A | G | 0.2564 | 0.3059 | 0.4223 |
|  |  | rs4605539 | T | C | 0.1618 | 0.1685 | 0.2618 |
|  |  | rs6768305 | G | C | 0.8551 | 0.8851 | 0.0519 |
|  |  | rs9855230 | G | A | 0.2148 | 0.2014 | 0.8964 |
|  |  | rs7646362 | A | G | 0.5556 | 0.5408 | **0.0353** |
|  |  | rs11429228 | N | C | 0.2634 | 0.2377 | 0.6270 |
|  |  | rs75581931 | A | G | **0.0289** | **0.0314** | 0.3670 |
|  |  | rs80118777 | G | T | **0.0397** | **0.0424** | 0.4000 |
|  |  | rs13320442 | G | A | 0.4083 | 0.3821 | 0.6923 |
|  |  | rs79062861 | T | C | 0.4138 | 0.3955 | 0.5989 |
|  |  | rs7623988 | G | C | 0.2163 | 0.0989 | 0.4610 |
|  |  | rs9836710 | G | A | 0.5561 | 0.5394 | 0.9062 |
|  |  | rs11921149 | A | G | 0.6827 | 0.6245 | 0.9969 |
|  |  | rs7619937 | A | G | 0.3146 | 0.4858 | 0.3488 |
|  |  | rs4579012 | T | C | 0.4508 | 0.3458 | 0.8893 |
|  |  | rs7642530 | A | G | 0.9460 | 0.9413 | 0.7910 |
|  |  | rs6793277 | G | T | 0.9254 | 0.8141 | 0.4211 |
|  |  | rs4522762 | C | T | 0.3738 | 0.3967 | 0.3497 |
|  |  | rs13434166 | G | A | 0.2506 | 0.2902 | 0.1833 |
|  |  | rs80303166 | G | A | 0.9422 | 0.9351 | 0.8289 |
|  |  | rs4566532 | G | T | 0.4301 | 0.2379 | 0.7397 |
|  |  | rs4637287 | G | C | 0.9382 | 0.9709 | 0.4141 |
|  |  | rs6776363 | C | G | 0.6791 | 0.5584 | 0.8197 |
|  |  | rs12492549 | G | C | 0.8062 | 0.3429 | 0.3687 |
|  |  | rs73124286 | T | G | 0.3997 | 0.4980 | 0.4735 |
| *GCKR* | 2 | rs1260326 | T | C | 0.7043 | 0.4710 | 0.1766 |
|  |  | rs4425043 | A | G | 0.8154 | 0.7894 | 0.7452 |
|  |  | rs780092 | G | A | 0.4254 | 0.2791 | 0.8397 |
| *PPARG* | 3 | rs73021485 | T | G | 0.3897 | 0.4115 | 0.5949 |
|  |  | rs6782178 | T | C | 0.6660 | 0.6671 | 0.8339 |
|  |  | rs2960422 | A | G | 0.6551 | 0.5301 | 0.9968 |
|  |  | rs73023314 | A | C | 0.4493 | 0.4671 | 0.4358 |
|  |  | rs12636461 | A | G | 0.8573 | 0.6143 | 0.8084 |
|  |  | rs10510411 | A | G | 0.8996 | 0.5625 | 0.3715 |
|  |  | rs12490265 | A | G | 0.6796 | 0.6616 | 0.6919 |
|  |  | rs10510418 | C | A | 0.4876 | 0.4687 | 0.8826 |
|  |  | rs1801282 | G | C | 0.5367 | 0.5378 | 0.8354 |
|  |  | rs4135247 | A | G | 0.7237 | 0.9311 | 0.4525 |
|  |  | rs13306745 | T | G | 0.7472 | 0.7221 | 0.4805 |
|  |  | rs2972162 | T | C | 0.6607 | 0.4463 | 0.7615 |
|  |  | rs4135268 | G | C | 0.8922 | 0.8950 | 0.7789 |
|  |  | rs4135275 | G | A | 0.7982 | 0.4739 | 0.7556 |
|  |  | rs4135283 | A | C | 0.7690 | 0.7132 | 0.9056 |
|  |  | rs117209672 | A | C | 0.2445 | 0.2379 | 0.9519 |
|  |  | rs1152001 | G | A | 0.6606 | 0.6743 | 0.5149 |
|  |  | rs3856806 | T | C | 0.7126 | 0.9827 | 0.1492 |
|  |  | rs1152003 | C | G | 0.3261 | 0.2804 | 0.6192 |
|  |  | rs9833097 | A | G | NA | NA | 0.6915 |
|  |  | rs17819328 | T | G | 0.1880 | 0.1919 | 0.4496 |
|  |  | rs78287138 | T | C | 0.3703 | 0.4693 | **0.0314** |
|  |  | rs9809905 | G | T | 0.9902 | 0.9929 | 0.8922 |
|  |  | rs4256108 | A | G | NA | NA | 0.9973 |
|  |  | rs11917039 | C | G | NA | NA | 0.8380 |
|  |  | rs709166 | A | G | 0.4714 | 0.4613 | 0.3010 |

A1 = minor allele, A2 = major allele, Chr = chromosome, MMSE = Mini-Mental State Examination.

P values of < 0.05 are shown in bold.

Analysis was obtained after adjustment for covariates including age, gender, and education.
