# Supplementary material for: The ADAMTS9 gene is associated with cognitive aging in the elderly in a Taiwanese population
Source: PLoS One. 2017 Feb 22;12(2):e0172440. doi: 10.1371/journal.pone.0172440 (PMC5321460; doi:10.1371/journal.pone.0172440)
Supplement: S4 Table — (DOC) [file pone.0172440.s004.doc]

**S4 Table.** MAF in various ethnic populations for five tag SNPs in the *ADAMTS9* gene.

|  | | | | | **MAF** | | | | |
| --- | --- | --- | --- | --- | --- | --- | --- | --- | --- |
| **Gene** | **CHR** | **SNP** | **A1** | **A2** | **Taiwanese**^a^ | **British**^b^ | **Japanese**^b^ | **African Americans**^b^ | **Han Chinese**^b^ |
| *ADAMTS9* | 3 | rs73832338 | T | C | 0.2070 | 0.0549 | 0.2212 | 0.3115 | 0.1602 |
|  |  | rs9985304 | A | G | 0.4273 | 0.6429 | 0.4712 | 0.5164 | 0.5243 |
|  |  | rs4317088 | C | T | 0.4895 | 0.8571 | 0.5337 | 0.5984 | 0.6019 |
|  |  | rs9831846 | C | T | 0.4840 | 0.8516 | 0.5577 | 0.5574 | 0.6214 |
|  |  | rs76346246 | C | T | 0.0702 | 0.0000 | 0.0769 | 0.0082 | 0.0971 |

A1 = minor allele, A2 = major allele, Chr = chromosome, MAF = minor allele frequency.

^a^ Data from the present study.

^b^ Data from the 1000 Genomes Project ([http://www.1000genomes.org](http://www.1000genomes.org/)).
